# Genetic Variability of Complement Factor H Has Ethnicity-Specific Associations With Choroidal Thickness

**DOI:** 10.1167/iovs.64.2.10

**Published:** 2023-02-07

**Authors:** Beau J. Fenner, Hengtong Li, Alfred T. L. Gan, Young Seok Song, Yih Chung Tham, Jost B. Jonas, Ya Xing Wang, Ching Yu Cheng, Tien Yin Wong, Kelvin Y. C. Teo, Anna C. S. Tan, Qiao Fan, Chui Ming Gemmy Cheung

**Affiliations:** 1 Singapore National Eye Centre, Singapore; 2 Singapore Eye Research Institute, Singapore; 3 Department of Ophthalmology, Asahikawa Medical University, Asahikawa, Hokkaido, Japan; 4 Centre for Innovation & Precision Eye Health, Department of Ophthalmology, Yong Loo Lin School of Medicine, National University of Singapore, Singapore; 5 Institute of Molecular and Clinical Ophthalmology, Basel, Switzerland; 6 Beijing Institute of Ophthalmology, Beijing Tongren Hospital, Capital Medical University, Beijing, China; 7 School of Medicine, Tsinghua University, Beijing, China; 8 Center for Quantitative Medicine, Duke-NUS Graduate Medical School, Singapore

**Keywords:** retina, choroid, genetics, age-related macular degeneration, AMD, pachychoroid, polypoidal choroidal vasculopathy, risk allele

## Abstract

**Purpose:**

To identify genetic alleles associated with differences in choroidal thickness (CT) in a population-based multiethnic Asian cohort.

**Methods:**

A population-based multiethnic Asian cohort without retinal pathology was subjected to spectral-domain OCT (SD-OCT) and genotyping of risk alleles in *CFH*, *VIPR2*, *ARMS2*, and *CETP*. Subfoveal choroidal thickness (SFCT) values were assessed from SD-OCT, and associations with the risk alleles were determined for each cohort.

**Results:**

A total of 1045 healthy Asian individuals (550 Chinese, 147 Indians, 348 Malays) were prospectively enrolled in the study. Several *CFH* alleles (rs800292, rs1061170, and rs1329428) were associated with increased SFCT in Indians (+18.7 to +31.7 µm; *P* = 0.001–0.038) and marginally associated with decreased SFCT in Malays (−12.7 to −20.6 µm; *P* = 0.014–0.022). Haplotype analysis of *CFH* revealed variable associations with SFCT among races, with the H6 haplotype being associated with a 29.08-µm reduction in SFCT in the Chinese cohort (*P* = 0.02) but a 35.2-µm increase in SFCT in the Indian cohort (*P* < 0.001). Finally, subfield analysis of the Chinese cohort identified associations between the *CFH* risk allele rs1061170 and reduced CT in the nasal and superior sectors (−20.2 to −25.8 µm; *P* = 0.003–0.027).

**Conclusions:**

*CFH* variants are variably associated with CT among Asian ethnic groups. This has broad implications for the pathogenesis of common diseases such as age-related macular degeneration and central serous choroidopathy, the pathogenesis of which is associated with CT.

The human visual pathway begins at the level of the photoreceptor cells in an energy-intensive process that depends on metabolic support from the adjacent retinal pigment epithelium and the underlying choroidal circulation. Choroidal perfusion of the photoreceptors and retinal pigment epithelium varies throughout the retina and is highest under the macula where the choroidal complex is thickest, but it thins markedly with aging.^1^ The Beijing Eye Study (BES) demonstrated that subfoveal choroidal thickness (SFCT) declines by 4 µm per year of age, in addition to a decrease of 15 µm per diopter of myopia.^2^ Choroidal thickness (CT) is also influenced by other factors, including blood pressure, diurnal variation,^3^ and genetic factors. Previous heritability estimates of CT range from 40% in the Amish^4^ to as high as 76% in Koreans.^5^

Understanding the genetic basis of CT has become an area of intense interest in recent years due to its association with age-related macular degeneration (AMD) and related exudative maculopathies.^6^^–^^9^ In particular, typical neovascular AMD and early AMD have been associated with reduced CT, whereas pachychoroid diseases such as polypoidal choroidal vasculopathy (PCV) and central serous chorioretinopathy (CSC) are associated with increased CT.^10^^,^^11^ A recent population-based genome-wide association study (GWAS) study of healthy Japanese individuals uncovered two single nucleotide polymorphisms (SNPs)—namely, rs3753394 (near the *CFH* locus) and rs7782658 (within the *VIPR2* locus)—that associated with increased CT independently of age, gender, and axial length.^12^ Previous studies have also identified genetic variants near or within *CFH*, which encodes complement factor H (CFH), in association with changes in CT.^13^^–^^15^ The majority of our understanding of the genetic factors that influence CT comes from Japanese cohort studies.^12^^–^^14^ It remains unclear if the impact of these genetic factors, in particular *CFH* variants, similarly affects CT across ethnic groups.

In the current work, we examined the association between the abovementioned CT risk alleles with CT variations in a large multiethnic population-based cohort to determine whether they exerted similar effects on CT among ethnic groups.

## Methods

### Study Participants

**Figure 1.**
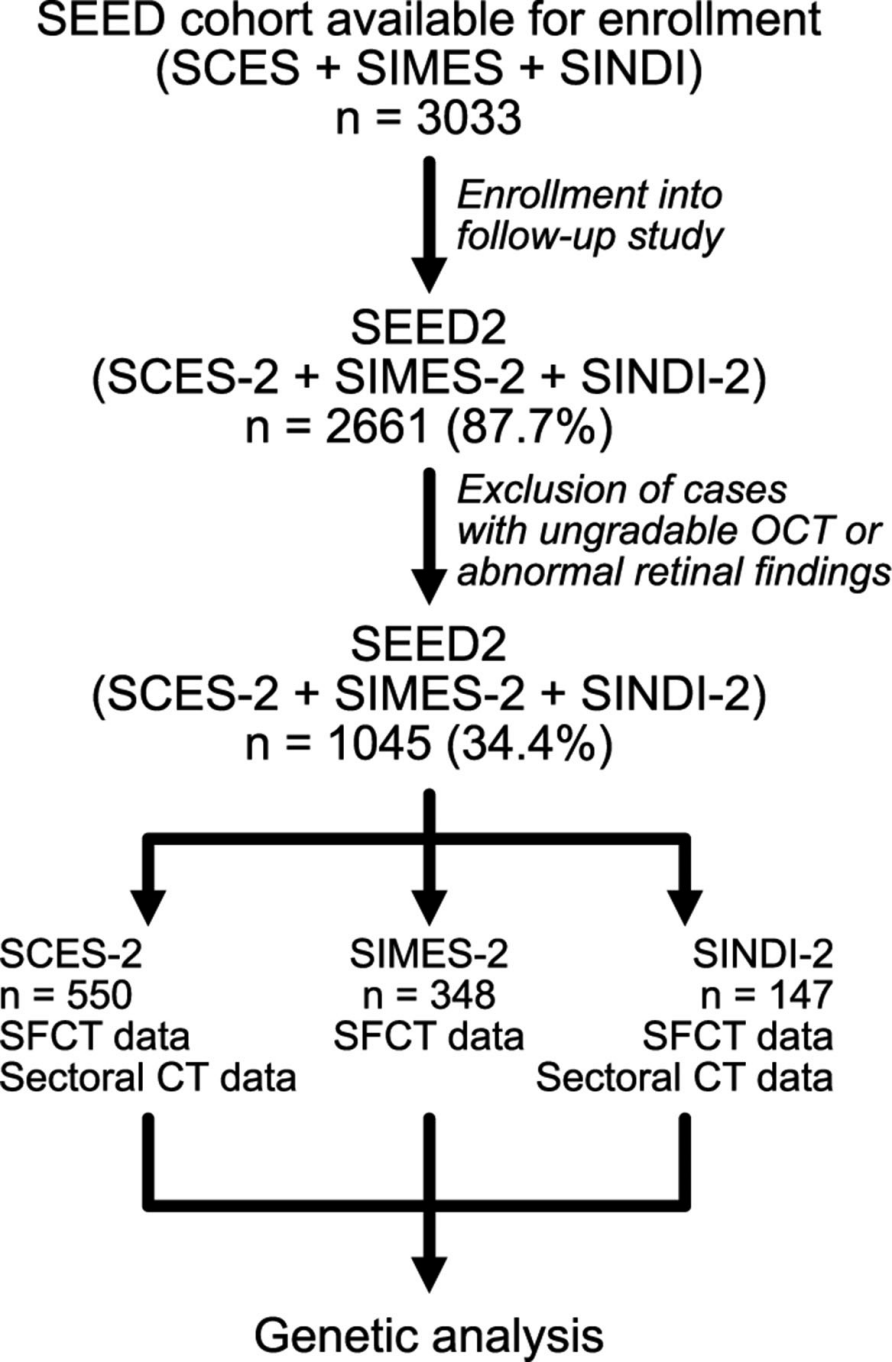
Flow chart summarizing patient enrollment for the current work. Baseline study enrollment for the Singapore Epidemiology of Eye Diseases (SEED) study yielded 3033 patients who were eligible for 6-year follow-up in the SEED-2 cohort studies (SCES-2, Singapore Chinese Eye Study 2; SIMES, Singapore Malay Eye Study 2; and SINDI-2, Singapore Indian Eye Study-2), of which 2661 participated and from which 1045 patients were eligible for analysis based on OCT quality and retinal findings. Data for sectoral CT thickness were available for the SCES-2 and SINDI-2 cohorts but not the SIMES cohort, whereas all cohorts had SFCT data available.

This analysis utilized retinal imaging of participants enrolled in the Singapore Chinese Eye Study (SCES), Singapore Indian Eye Study, and the Singapore Malay Eye Study as described in detail elsewhere.^16^ These studies were population-based epidemiological studies conducted in Singapore. A total of 4605 individuals were eligible based on age stratification, of which 3353 individuals (response rate of 72.8%) were eventually recruited and participated in the baseline study from 2009 to 2011. Of the 3033 eligible individuals originally enrolled in the population cohort studies (some participants dropped out for various reasons including demise, migration, and ill health, including cognitive and mobility impairment), 2661 participated in the 6-year follow-up studies and underwent spectral-domain optical coherence tomography (SD-OCT) imaging (response rate of 87.7%). Cases with poor-quality OCT images or evidence of macular pathology noted on OCT or color fundus photography were excluded. The process of patient enrolment is summarized in Figure 1. This work was approved by the SingHealth Centralized Institutional Review Board and adhered to the tenets of the Declaration of Helsinki. Written informed consent was obtained from each patient prior to involvement in the study.

**Figure 2.**
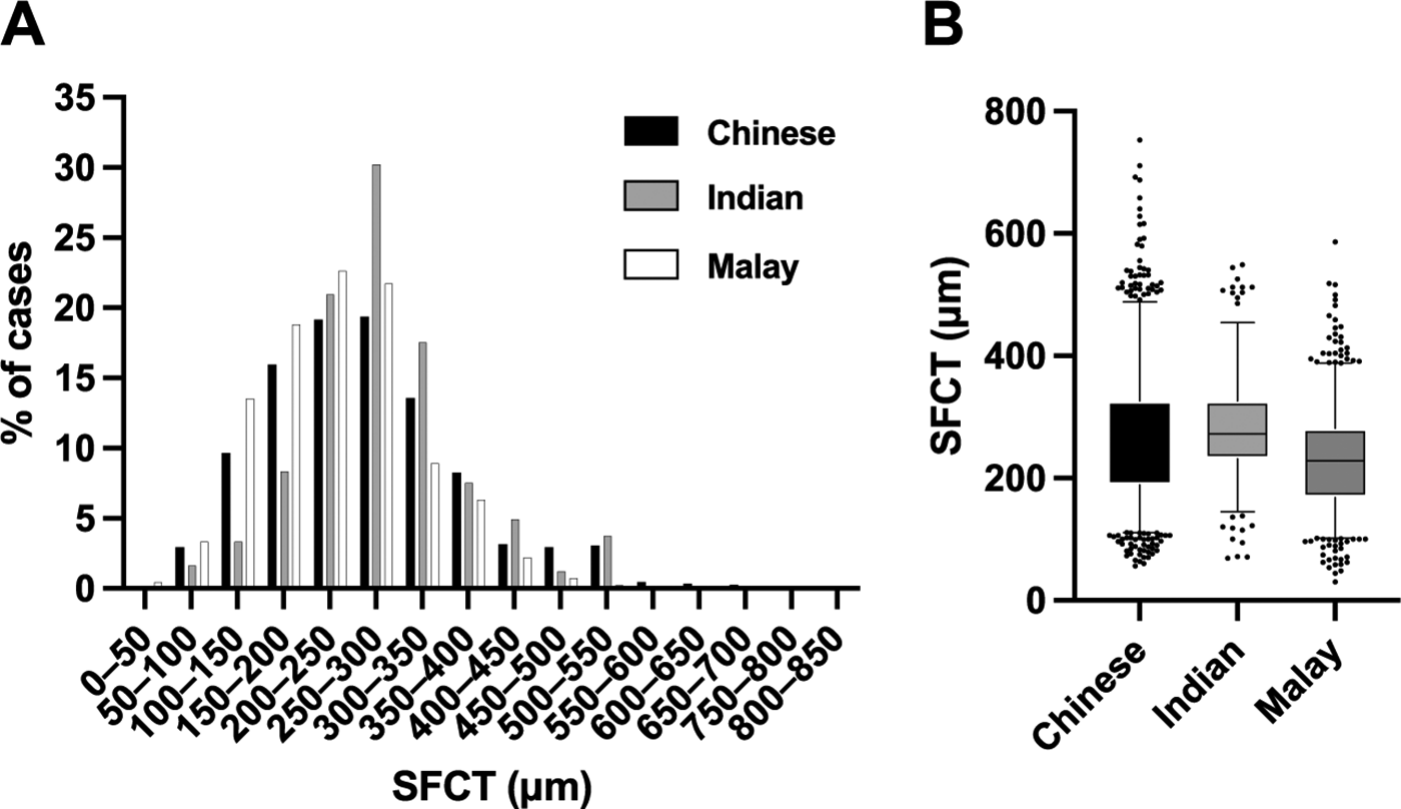
Distribution of CT among the different ethnic cohorts. (A) The proportion of cases with the indicated range of SFCT values is shown for each ethnic cohort. (B) Box-and-whisker plot with the *central line* of the boxes representing the median subfoveal CT (in µm); the *lower* and *upper edges* of the boxes are the first and third quartiles, respectively. The *error bars* show the 5th and 95th centile values. Individuals above and below the 5th and 95th centiles are plotted as individual points.

### Clinical Examination

Each participant underwent a standardized ocular examination as described in the SCES-2 study protocol.^16^^–^^18^ Briefly, each participant underwent subjective refraction by a trained optometrist to determine their spherical equivalent and best-corrected visual acuity. Intraocular pressure was measured with the Goldmann applanation tonometer (Haag-Streit, Köniz, Switzerland) prior to pupil dilation. Ocular biometry was performed to determine axial length using an IOLMaster 3 noncontact partial coherence interferometer (Carl Zeiss Meditec AG, Jena, Germany). Slit-lamp biomicroscopy was performed by study ophthalmologists using a Haag-Streit BQ-900 slit lamp.

### Determination of Choroidal Thickness

Study participants underwent macular SD-OCT with enhanced depth imaging modality (SPECTRALIS; Heidelberg Engineering, Heidelberg, Germany) after pupil dilation with tropicamide 1% and phenylephrine hydrochloride 2.5%. The imaging protocol was comprised of 31 horizontal 9-mm raster B-scans centered on the fovea and spanning 30° × 30° of the macula. Each B-scan was averaged 75 times during acquisition. Images were acquired in high-speed mode. Scans with a signal strength of ≥20 were included in the analysis. Images with motion artifact, that were poorly focused, or that had an obscured choroidal–scleral interface were excluded from the analysis. The SFCT was measured by two trained graders using the built-in Heidelberg SPECTRALIS software caliper tool, and the measurements recorded were averaged to obtain the final measurement. Graders were consultant ophthalmologists with fellowship training in retina. In case of a discrepancy exceeding 10% of the mean SFCT, the measurements were adjudicated by a third observer (CMGC) who gave a final measurement. Mean CT was measured by averaging the CT within each sector (slab analysis of the inner and outer rings of the superior, inferior, nasal, temporal, and central 1-mm zones) of the standard Early Treatment Diabetic Retinopathy Study grid centered on the fovea. This was done using the SPECTRALIS Heyex SP-X 6.4.8.116 software module, as described previously.^18^ These scan data were available only for the Chinese and Indian cohorts. OCT imaging was performed during normal clinic operating hours of 8.30 AM to 5.30 PM, and the imaging of patients of each ethnic cohort was evenly distributed throughout these hours.

### SNP Selection and Genotyping, Quality Control

We identified eight SNPs located within or surrounding four different loci (*CFH*, *ARMS2*, *VIPR2*, and *CETP*) that were found to associate with SFCT positively or negatively in previous studies (Table 1).^12^^–^^15^ Allele frequencies for the chosen SNPs are shown in Supplementary Table S1. The association of each risk allele with SFCT was assessed via multivariable analysis that adjusted for differences in age, gender, axial length, and refractive error and with clustering by individual. Whole genome makers were genotyped using the Human OmniExpress BeadChip system (Illumina, San Diego, CA, USA). Stringent quality-control filters were applied to samples and SNP markers. SNPs showing deviation from Hardy–Weinberg equilibrium (*P* < 10^−6^) and with missingness > 5%, were removed. Poorly genotyped samples (missingness > 5%) were excluded from the analysis. Identity-by-state information was determined using KING 2.2.4,^19^ and individuals with the lower call rate were excluded for inferred pairs of first-degree relatives. Principal component analysis was conducted to identify subpopulation structure using EIGENSTRAT 2.0.^20^ Additional quality-control filters were applied to exclude SNPs with >1% missingness if minor allele frequency (MAF) was less than 5%.^21^

### Inclusion and Exclusion Criteria

Inclusion and exclusion criteria were essentially the same as those described in our previous work.^18^ Briefly, we excluded eyes with retinal disease, previous retinal surgery, or laser treatment because these conditions have been associated with alterations in CT. The presence of retinal diseases was based on the grading of fundus photographs and OCT, as described in detail previously.^18^ Eyes with other ocular conditions, such as refractive error, were included if the quality of OCT image enabled accurate grading.

### Statistical Analysis

We performed association tests under an additive model for each genotyped SNP, using SFCT as a quantitative outcome. We used data for both eyes for single variant analysis to boost the statistical power. Using Stata 16 (StataCorp, College Station, TX, USA), a generalized estimating equation (GEE) regression analysis was conducted to estimate the beta effects and 95% confidence intervals (CIs); the analysis accounted for age, gender, top three principal components, and clustering by individuals. We further accounted for axial length and refractive error in the GEE model, yielding similar results. Thus, we presented the results from the full model. Haplotype analyses and Gaussian linear regression were performed using the R package haplo.stats.^18^ Only right-eye data were used for haplotype analyses, adjusting for age, gender, axial length, refractive error, and top three principal components. The haplotype that did not contain any risk alleles was set as the reference. Rare haplotypes with frequencies less than 1% in all ethnic groups were categorized in one haplotype group (rare H). For the single variant analysis, the threshold *P* value of 6.25 × 10^−3^, after Bonferroni correction, was set for the significance in the association analysis. The significance threshold for the four CFH variants across four subfields was set at *P* = 3.13 × 10^−3^.

## Results

### Characteristics of Study Subjects

We included 550 Chinese, 147 Indian, and 348 Malay subjects from the Singapore Epidemiology of Eye Diseases population-based cohorts. Gender proportions were similar among cohorts (Table 2), whereas the Chinese cohort was younger (mean age, 58.7 ± 6.1 years) and the Malay cohort was older (mean age, 62.7 ± 8.4 years) than the Indian cohort (mean age, 60.4 ± 6.9 years). The Chinese cohort additionally had a significantly higher axial length and a refractive error of a more minus spherical equivalent than the Indian and Malay cohorts (Table 2). Single-point caliper measurements of the SFCT on SD-OCT imaging (Fig. 2) revealed that the mean SFCT was highest in the Indian cohort (282.4 ± 87.2 µm) and lowest in the Malay cohort (231.9 ± 86.9 µm).

**Table 1.** SNPs Used for Analysis in the Current Work

						Reported Associations for Effect Allele				
SNP Name	Chromosomal Location	Gene	Consequence	Effect Allele^*^	Other Allele	AMD^†^	PCV	CSC	SFCT	References
rs800292	chr1:196642233	*CFH*	Missense variant	G	A	+(G)	+(G)	+(A)	+(A)	Hosoda et al.,^12^ Hageman et al.,^37^ Liu et al.,^38^ Cipriani et al.,^39^ Kondo et al.^40^
rs1061170	chr1:196659237	*CFH*	Missense variant	C	T	+(C)	+(C)	+(T)	+(T)	Ryoo et al.,^15^ Kondo et al.,^40^ Haines et al.,^41^ Edwards et al.,^42^ Lima et al.,^43^ Mohabati et al.^44^
rs1329428	chr1:196702810	*CFH*	Intron variant	C	T	+(C)	—	+(T)	+(T)	Yoneyama et al.,^13^ Cipriani et al.,^39^ Klein et al.,^45^ Kiraly et al.^46^
rs61818925	chr1:196815450	*CFH*	No consequence	G	T	+(G)	—	—	—	Cipriani et al.,^39^ Pappas et al.^47^
rs3793217	chr7:158848821	*VIPR2*	Intron variant	G	A	—	—	+(G)	+(G)	Hosoda et al.^12^
rs7782658	chr7:158858007	*VIPR2*	Intron variant	A	G	—	—	—	+(A)	Hosoda et al.^12^
rs10490924	chr10:124214448	*ARMS2*	Missense variant	T	G	+(T)	+(T)	+(G)	+(G)	Yoneyama et al.,^13^ Kondo et al.,^40^ Klein et al.,^45^ Jakobsdottir et al.^48^
rs3764261	chr16:56993324	*CETP*	No consequence	A	C	+(A)	+(A)	—	—	Liu et al.,^38^ Chen et al.^49^

* Effect allele associated with increased risk of AMD, except for rs3793217 and rs7782658 in *VIPR2*.

† AMD was variously defined as neovascular AMD or both neovascular and non-neovascular AMD in the references cited.

**Table 2.** Baseline Characteristics of the Singaporean Ethnic Cohorts Used in the Current Study

	All Cohorts	Chinese Cohort	Indian Cohort	Malay Cohort	*P*
Number of eyes included, *n*	1788	941	238	609	
Number of individuals included, *n*	1045	550	147	348	
Age (y), mean ± SD	60.3 ± 7.3	58.7 ± 6.1	60.4 ± 6.9	62.7 ± 8.4	<0.001^*^
Female gender, *n* (%)	537 (51.4)	282 (51.3)	74 (50.3)	181 (52.0)	0.941
Axial length (mm), mean ± SD	23.8 ± 1.1	24.0 ± 1.2	23.4 ± 0.9	23.5 ± 0.9	<0.001^†^
Spherical equivalent (diopters), mean ± SD	−0.13 ± 2.21	−0.62 ± 2.47	0.56 ± 1.78	0.34 ± 1.69	<0.001^†^
Subfoveal CT (µm), mean ± SD	257.4 ± 101.8	267.6 ± 110.6	282.4 ± 87.2	231.9 ± 86.9	<0.001^‡^
Mean CT (µm), mean ± SD	281.5 ± 72.2	280.6 ± 75.1	284.9 ± 58.9	NA^§^	0.446

Multiple comparisons of pairwise cohorts for each characteristic used a Bonferroni-corrected *P* value of 0.05/3 = 1.67E-2.

* Significant for all pairwise comparisons.

† Significantly different between Chinese and Indians and between Chinese and Malays.

‡ Significantly different between Malays and Chinese and between Malays and Indians.

§ Scan modes required to calculate mean CT were not available at the time of imaging the Malay cohort.

### Association of AMD Risk Alleles with CT

**Figure 3.**
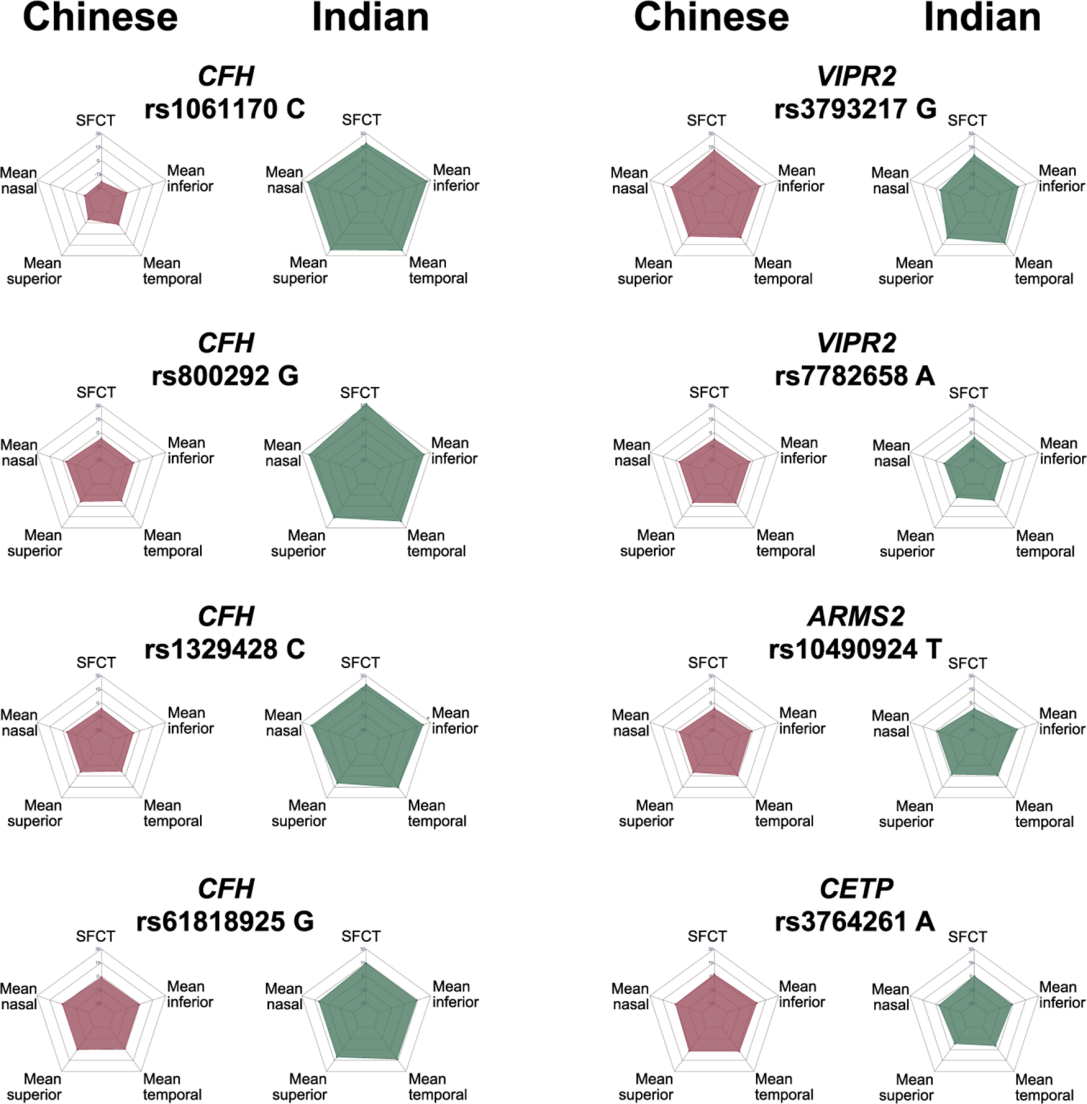
Associations of risk allele dose on CT in macular sectors comparing Chinese and Indian study populations. For this analysis, data were available for the Chinese and Indian cohorts only.

Risk alleles rs800292, rs1061170, rs1329428, and rs10922109 in *CFH* and rs3793217 in *VIPR2* were marginally associated with a decrease in SFCT of between 12.7 and 20.6 µm per allele dose in the Malay cohort (*P* = 0.014–0.037) (Table 3). Conversely, these same risk alleles in *CFH* were significantly associated with an increase in SFCT of between 18.7 and 31.7 µm per allele dose among the Indian cohort (*P* = 0.001–0.038) (Table 3). We additionally compared the association of SFCT with the same risk alleles in the previously described Beijing Eye Study (BES) cohort of ethnic Chinese and found similar results, both as an individual BES cohort and when combined with the Singapore cohort as a meta-analysis (Supplementary Table S2). We further analyzed the Chinese and Indian cohorts to identify associations between CT in different macular OCT subfields and the risk alleles (Fig. 3 and Supplementary Table S3). With this approach, we identified an association between the *CFH* rs1061170 C allele and reduced CT in the nasal and superior subfields among Chinese (−20.3 to −25.5 µm; *P* = 0.002–0.027). Findings for the Indian cohort were similar for both the SFCT and the macular subfield CT in terms of association with the *CFH* risk alleles. We also assessed the difference in CT for each macular sector for each effect allele. Although the *VIPR2*, *ARMS2*, and *CETP* alleles exhibited similar trends in association for both the Chinese and Indian cohorts (Fig. 3; scan data available only for Chinese and Indian cohorts), there were differences in CT for each sector when comparing the four *CFH* alleles. In particular, the rs1061170 and rs800292 alleles were associated with lower CT for the Chinese cohort and higher CT for the Indian cohort (Fig. 3). Adjustment for the time of OCT acquisition (AM or PM) did not have a significant impact on these findings, indicating that they were not the result of diurnal variability in CT caused by differences in the time of OCT imaging among the different ethnic cohorts (Supplementary Table S4).

**Table 3.** Association of AMD and CSC Risk Allele Doses With SFCT in the Three Ethnic Cohorts

		All Cohorts (*N* = 1045; 1788 Eyes)		Chinese (*N* = 550; 941 Eyes)		Indian (*N* = 147; 238 Eyes)		Malay (*N* = 348; 609 Eyes)	
SNPs	Effect Allele/Other Allele	β (95% CI)^†^	>*P*^‡^	>β (95% CI)^†^	>*P*^‡^	>β (95% CI)^†^	>*P*^‡^	>β (95% CI)^†^	>*P*^‡^
*CFH*
rs800292	G/A	−4.7 (−12.5 to 3.0)	0.228	−6.6 (−18.1 to 5.0)	0.266	31.7 (12.6 to 50.9)	0.001^*^	−13.0 (−23.4 to −2.7)	0.014^*^
rs1061170	C/T	−4.1 (−15.3 to 7.1)	0.474	−23.6 (−50.2 to 3.0)	0.082	18.7 (1.1 to 36.3)	0.038^*^	−20.6 (−37.3 to −3.9)	0.016^*^
rs1329428	C/T	−5.1 (−12.5 to 2.3)	0.177	−6.7 (−17.6 to 4.2)	0.228	19.8 (3.0 to 36.6)	0.021^*^	−12.7 (−23.5 to −1.9)	0.022^*^
rs61818925	G/T	−2.2 (−9.2 to 4.9)	0.547	−1.7 (−11.9 to 8.4)	0.736	14.2 (−3.6 to 32.0)	0.117	−9.7 (−20.7 to 1.3)	0.083
*VIPR2*									
rs3793217	G/A	3.0 (−9.3 to 15.2)	0.637	11.2 (−4.8 to 27.3)	0.170	5.7 (−29.2 to 40.5)	0.748	−17.9 (−34.8 to −1.1)	0.037^*^
rs7782658	A/G	−5.8 (−15.0 to 3.4)	0.218	−7.4 (−20.8 to 6.0)	0.279	−6.0 (−27.4 to 15.4)	0.581	−5.2 (−19.0 to 8.6)	0.461
*ARMS2*									
rs10490924	T/G	−4.2 (−11.2 to 2.8)	0.242	−6.5 (−16.3 to 3.4)	0.197	−6.9 (−23.1 to 9.4)	0.404	0.8 (−10.1 to 11.6)	0.892
*CETP*									
rs3764261	A/C	6.8 (−2.4 to 16.1)	0.147	1.9 (−12.6 to 16.4)	0.792	−0.0 (−18.2 to 18.1)	0.996	7.2 (−8.4 to 22.9)	0.365

* *P* values between 0.05 and 6.25E-3 were deemed marginally significant.

† Beta coefficient is the increase in mean SFCT (µm) for each effect allele increase and is derived from linear regression of SFCT against genotypes, adjusted for age, gender, axial length, and refractive error, with clustering by individual. The results are similar from the model without adjusting for axial length and refractive error.

‡ Bonferroni-corrected *P* of 0.05/8 = 6.25E-3 was used as a cutoff for significance.

### Varying Haplotype Effects in Ethnic Groups

In view of the association between the *CFH* alleles and SFCT, we performed a haplotype analysis to determine if combinations of the *CFH* risk alleles (rs800292, rs1061170, rs1329428, and rs61818925) were associated with greater increases or decreases in SFCT (Table 4). The haplotype association with SFCT varied significantly among ethnic groups. The H6 haplotype, comprised of AMD risk alleles, occurred in only 3.6% of Chinese and was associated with a 29-µm reduction in SFCT, whereas the same haplotype was present in 28.2% of Indians and was associated with a 35-µm increase in SFCT. Haplotype H3 was associated with an increase in SFCT of nearly 50 µm among Indians but was not significantly associated with SFCT in the Chinese or Malays. Several low-frequency haplotypes (MAF < 2%) exhibited unique associations in the Chinese/Malay or Indian cohorts (*P* < 0.001). The H7 haplotype was seen in 1.9% of Chinese and 1.5% of Malays, where it was associated with a 24.9-µm (*P* < 0.001) and 13.9-µm (*P* < 0.001) increase in SFCT but was rarely seen (0.7%) in Indians. Conversely, the H10 haplotype occurred in 2.9% of Indians and was associated with a 46.4-µm increase in SFCT but was rarely seen in Chinese (0.3%) and Malays (0.4%).

**Table 4.** Haplotype Analysis of the *CFH* Gene in Three Populations

					Chinese			Indian			Malay		
Haplotype	rs800292	rs1061170	rs1329428	rs61818925	Frequency	Beta (95% CI)	*P* Value^*^	Frequency	Beta (95% CI)	*P* Value^*^	Frequency	Beta (95% CI)	*P* Value^*^
H1	A	T	T	T	0.324	1 (Ref.)	1 (Ref.)	0.193	1 (Reference)	1 (Ref.)	0.314	1 (Ref.)	1 (Ref.)
H2	G	T	C	G	0.337	−6.89 (−19.08 to 5.3)	0.27	0.083	−6.93 (−40.22 to 26.36)	0.68	0.356	−11.95 (−25.68 to 1.79)	0.09
H3	G	T	C	T	0.154	−7.08 (−23.87 to 9.71)	0.41	0.074	49.09 (15.76 to 82.41)	3.89E−03^*^	0.077	−17.97 (−43.53 to 7.6)	0.17
H4	A	T	T	G	0.065	−1.22 (−24.66 to 22.22)	0.92	0.080	9.49 (−24.97 to 43.96)	0.59	0.045	16.58 (−17.02 to 50.17)	0.33
H5	G	T	T	T	0.044	−1.23 (−23.63 to 21.18)	0.91	0	NA^†^	NA^†^	0.063	−4.36 (−30.43 to 21.7)	0.74
H6	G	C	C	G	0.036	−29.08 (−53.89 to −4.26)	0.02	0.282	35.25 (12.96 to 57.53)	1.93E-03^*^	0.083	−20.22 (−43.69 to 3.26)	0.09
H7	A	T	C	G	0.019	24.97 (23.73 to 26.2)	<1E-100^*^	0.007	NA^†^	NA^†^	0.015	13.9 (10.57 to 17.22)	2.22E-16^*^
H8	G	T	T	G	0.012	20.06 (15.92 to 24.2)	2.17E-21^*^	0.234	15.04 (−7.63 to 37.71)	0.19	0.025	−29.46 (−33.26 to −25.67)	2.37E-52^*^
H9	A	T	C	T	0.007	NA^†^	NA^†^	0	NA^†^	NA^†^	0.011	−26.4 (−33.22 to −19.58)	3.31E-14^*^
H10	G	C	C	T	0.003	NA^†^	NA^†^	0.029	46.39 (23.12 to 69.67)	9.37E-05^*^	0.004	NA^†^	NA^†^
H11	A	C	C	G	0	NA^†^	NA^†^	0.016	−6.98 (−12.1 to −1.85)	7.68E-03^*^	0.007	NA^†^	NA^†^
Rare H^‡^	NA^†^	NA^†^	NA^†^	NA^†^	0.011	−22.22 (−25.04 to −19.4)	1.13E-53^*^	0.011	93.7 (91.76 to 95.65)	<1E-100^*^	0.010	15.56 (11.98 to 19.15)	1.82E-17^*^

Haplotype analyses were performed with haplotype H1 comprising the protective alleles for AMD as reference.

* Analyses were adjusted for age, gender, top three principal components, axial length, and spherical equivalence. Significant associations after Bonferroni correction at *P* values of 0.05/8 = 6.25E-3 or 0.05/9 = 5.56E-3.

† Haplotypes with frequency less than 1% in a specific population were excluded from analysis.

‡ Rare haplotypes (Rare H) with frequency less than 1% across all populations were clustered as one haplotype group. Data for rare haplotypes includes an accumulation of alleles that could be either allele.

### Heterogeneity of Genetic Effects by Ethnicity

To further explore our observation that SFCT was differentially associated with the risk alleles among the different ethnic cohorts, we performed pairwise interaction analyses for each ethnic group. A significant difference was identified for risk alleles in *CFH* when comparing the Chinese and Indian cohorts, whereas no significant interaction was found for the *VIPR2*, *ARMS2*, and *CETP* alleles. Similar interactions in the *CFH* risk alleles were observed between the Indian and Malay cohorts. A comparison of the Malay and Chinese cohorts demonstrated a significant interaction only for the risk allele rs7782658 in *VIPR2* (Supplementary Table S5).

## Discussion

Our study showed that genetic variants of *CFH* were differentially associated with CT in a multiethnic Asian cohort. At the individual risk allele level, the presence of *CFH* alleles rs800292, rs1061170, rs1329428, or rs10922109 was associated with a significant increase in CT among the Indian cohort, whereas the same alleles were associated with a significant decrease in thickness among Malay individuals. Additionally, we also found that the *VIPR2* rs3793217 allele was associated with reduced SFCT in a Malay cohort but not in the Chinese or Indian cohorts. We also demonstrated that specific *CFH* risk allele haplotypes were differentially associated with increased CT among different ethnic cohorts. Importantly, these associations were independent of age, gender, axial length, or refractive error. These findings suggest that CT is influenced by genetic variability, and to our knowledge this is the first report of the impact of *CFH* risk alleles on CT in a multiethnic study cohort.

Previous GWAS analysis of a large healthy Japanese cohort identified SNPs of *CFH* (rs800292) and *VIPR2* (rs3793217) that were associated with SFCT.^12^ In our ethnic Chinese cohort and the BES cohorts we did not observe a significant association between these SNPs and SFCT, but we did find a significant association between rs800292 and SFCT among the Indian and Malay cohorts. Interestingly, this association was in opposite directions between these cohorts, with increased SFCT being seen in the Indian cohort and decreased SFCT observed in the Malay cohort. For the *VIPR2* allele rs3793217, we only observed significant association with SFCT for the Malay cohort, and again this was in the opposite direction from that seen in the previously published Japanese cohort.^12^

Previous reports on Japanese cohorts identified an association between AMD risk alleles *CFH* rs1329428 (C allele) and *ARMS2* rs10490924 (T allele) and decreased CT.^13^^,^^14^ In contrast, the rs1329428 risk allele was marginally associated with increased CT for our Indian cohort, whereas the same allele was associated with marginally decreased CT among the Malay cohort, with no association being found for the Chinese cohort. We also did not observe any significant association between *ARMS2* rs10490924 and CT. Our additional analysis of a large cohort of individuals from the BES^2^ did not demonstrate an association between individual risk alleles and CT, like our Chinese cohort. This provides evidence that the influence of these risk alleles on CT is distinct in different Asian populations.

The contrasting associations between CT and *CFH* alleles among the three different ethnic groups may be the result of differences in the genetic backgrounds of the ethnic groups. For the Indian cohort, the G allele of rs800292 and the C allele of rs1329428 in combination (haplotypes H3, H6, and H10) associate with significant increases in CT (35–49 µm, depending on haplotype), whereas a weak or strong negative association with CT was seen for these alleles in the Chinese and Malay cohorts. The H7 and H8 haplotypes were associated with increased CT in our Chinese cohort, with the alleles in common being rs1061170 (T allele) and rs6181825 (G allele). Unlike the Indian cohort, these alleles alone were not sufficient to explain the observed effect, as other haplotypes (H2 and H4) were not associated with changes in CT. Despite this, it seems likely from these data that ethnic background has distinct effects on the interplay between CT and the previously characterized genetic polymorphisms.

The association between genetic variants in the *CFH* and *VIPR2* genes and CT prompts consideration of the possible underlying physiological mechanisms. The relationship of both the factor H and VIPR2 proteins to CT appears to be related to their influence on vascular dilatation. Although factor H was initially characterized as a complement regulator,^22^^,^^23^ it was subsequently appreciated that it also functioned as an adrenomedullin-binding protein.^24^^,^^25^ Adrenomedullin is a vasodilator that stimulates increased choroidal blood flow in humans,^26^^,^^27^ and CFH (also known as adrenomedullin-binding protein) may play a role in potentiating this effect.^28^ Variability in the *CFH* gene might also be expected to influence the effect of adrenomedullin on choroidal circulation and thickness. Similarly, the VIPR2 protein is a receptor for vasoactive intestinal peptide, a potent vasodilator, and variability in the *VIPR2* gene sequence may modulate CT.^29^^,^^30^

We acknowledge several important limitations of this work. First, we screened our study cohorts for the presence of previously characterized risk alleles, which precluded the possibility of identifying novel and potentially important markers for CT in our study population. A GWAS like that described by Hosoda and colleagues^12^ would be ideal for discovery of genetic variants associated with SFCT. The sample size for the current study was insufficient to detect genome-wide significance, although collaborative efforts are underway to perform this analysis. Second, it is well established that the measurement of CT is susceptible to diurnal variability. Previous estimates of the change in CT ranged from 13 to 33 µm over the course of a day,^3^^,^^31^^,^^32^ which is at least as much as the unit increase in SFCT associated with our examined SNPs. This variability could be reduced by limiting image acquisition to a specific time of day, although this would pose considerable logistical challenges for a large population-based cohort study such as ours. Our sensitivity analysis did not show any significant change to the results after adjusting for the general time of day (i.e., AM or PM) for image acquisition, although more granular analysis with the specific time of day could potentially disclose differences not captured by this analysis. Another limitation is that SFCT was only measured from a single B-scan and was used as the phenotype measures to determine genetic associations. It is possible that SFCT is not representative of the overall CT; hence, we performed additional analysis based on CT in different regions, although volumetric measurement of CT may render different associations. Finally, another potential limitation of our study is the lack of younger age groups in our study cohort. CT has been observed to decline with advancing age,^33^^–^^36^ and it remains unclear how the association of the studied risk alleles with SFCT changes during the life of an individual. Interestingly, data from a large Chinese cohort suggested that SFCT only declined after the age of 60^34^; thus, the older age of our cohort with a mean age of approximately 60 years may not have influenced our findings.

In conclusion, our results reveal that an ethnic component underpins the association of CT with previously characterized AMD and CSC risk alleles. This may have implications for the manifestations of these conditions seen among different ethnic groups in clinical practice. Future work may focus on the relevance of these risk alleles and these newly defined risk haplotypes among individuals for exudative macular diseases, including typical neovascular AMD, PCV, and CSC in different ethnic groups.

## Supplementary Material

Supplement 1
