# Supplementary material for: Genetic Variability of Complement Factor H Has Ethnicity-Specific Associations With Choroidal Thickness
Source: Invest Ophthalmol Vis Sci. 2023 Feb 7;64(2):10. doi: 10.1167/iovs.64.2.10 (PMC9919691; doi:10.1167/iovs.64.2.10)
Supplement: Supplement 1 [file iovs-64-2-10_s001.pdf]

Supplementary Table 1. Allele frequencies for the study SNPs among the different Singaporean ethnic cohorts in the current study.

SNPs	Chr:BP	Allele	All cohorts (n = 1045)	Chinese cohort (n = 550)	Indian cohort (n = 147)	Malay cohort (n = 348)	Significance (p)[a]
CFH							
rs800292	1:196642233	G	0.610	0.585	0.704	0.608	0.007[b,d]
rs1061170	1:196659237	C	0.098	0.039	0.330	0.093	<0.001 [b,c,d]
rs1329428	1:196702810	C	0.545	0.555	0.490	0.553	0.258
rs61818925	1:196815450	G	0.522	0.468	0.701	0.532	<0.001[b, c, d]
VIPR2							
rs3793217	7:158848821	G -	0.106	0.124	0.082	0.089	0.108
rs7782658	7:158858007	A -	0.192	0.191	0.211	0.187	0.752
ARMS2							
rs10490924	10:124214448	T -	0.411	0.432	0.337	0.411	<0.001[b, c, d]
CETP							
rs3764261	16:56993324	A -	0.174	0.162	0.313	0.135	<0.001[b,d]

[a] Chi–squared test comparing number of effect alleles (0, 1 or 2) between ethnic groups. A Bonferroni corrected p value cutoff of 0.05/8=6.25E-3 was used to determine statistical significance.

Multiple comparisons among three cohorts used a p value cutoff of 0.05/3=0.17E-2.

[b] Significantly different between Chinese and Indian cohorts

[c] Significantly different between Chinese and Malay cohorts

[d] Significantly different between Malays and Indian cohorts

Supplementary Table 2. Association of AMD and CSC risk alleles with subfoveal choroidal thickness (SFCT) among individuals from the current study and from those described previously in the Beijing Eye Study (BES) cohort. Meta-analysis of both cohorts using inverse variance was also performed.

SNP	Risk allele/ref allele	SCES (550 individuals)		BES (982 individuals)		Meta analysis	
		β (95% CI)[a]	p	β (95% CI)[a]	p	β (95% CI)[a]	p
CFH							
rs800292	G/A	-6.6 (-18.1 to 5.0)	0.266	-5.3 (-13.2 to 2.6)	0.186	-5.7 (-12.2 to 0.8)	0.085
rs1061170	C/T	-23.6 (-50.2 to 3.0)	0.082	5.7 (-10.3 to 21.6)	0.486	-2.2 (-15.9 to 11.5)	0.754
rs1329428	C/T	-6.7 (-17.6 to 4.2)	0.228	-3.5 (-11.3 to 4.2)	0.375	-4.6 (-10.8 to 1.7)	0.151
rs61818925	G/T	-1.7 (-11.9 to 8.4)	0.736	3.2 (-4.5 to 11.0)	0.412	1.4 (-4.7 to 7.5)	0.653
VIPR2							
rs3793217	G/A	11.2 (-4.8 to 27.3)	0.170	-1.3 (-11.3 to 8.8)	0.806	2.4 (-6.2 to 11.1)	0.581
rs7782658	A/G	-7.4 (-20.8 to 6.0)	0.279	1.8 (-7.3 to 10.9)	0.697	-1.1 (-8.6 to 6.4)	0.775
ARMS2							
rs10490924	T/G	-6.5 (-16.3 to 3.4)	0.197	-6.7 (-14.8 to 1.3)	0.101	-6.6 (-12.8 to -0.4)	0.037
CETP							
rs3764261	A/C	1.9 (-12.6 to 16.4)	0.792	0.1 (-10.3 to 10.5)	0.983	0.6 (-7.2 to 8.4)	0.876

[a]Beta coefficient is the increase in mean SFCT (μm) for every one risk allele increase and is derived from linear regression of SFCT against allele frequency, adjusted for age, gender, axial length and refractive error, with clustering by individual. P value threshold after the Bonferroni correction is 0.05/8=6.25E-3.

Supplementary Table 3. Association of AMD and CSC risk alleles with variation of subfields of choroidal thickness in Chinese and Indian populations.

**Index**	**Subfield of CT**	**rs800292_G**		**rs1061170_C**		**rs1329428_C**		**rs61818925_G**		**rs3793217_G**		**rs7782658_A**		**rs10490924_T**		**rs3764261_A**	
		**beta (95% CI)**	**p**	**beta (95% CI)**	**p**	**beta (95% CI)**	**p**	**beta (95% CI)**	**p**	**beta (95% CI)**	**p**	**beta (95% CI)**	**p**	**beta (95% CI)**	**p**	**beta (95% CI)**	**p**
**Chinese (941 eyes, 550 individuals)**																	
1	CT of SFCT (center)	–6.6 (–18.1, 5.0)	0.266	–23.6 (–50.2, 3.0)	0.082	–6.7 (–17.6, 4.2)	0.228	–1.7 (–11.9, 8.4)	0.736	11.2 (–4.8, 27.3)	0.170	–7.4 (–20.8, 6.0)	0.279	–6.5 (–16.3, 3.4)	0.197	1.9 (–12.6, 16.4)	0.792
2	CT at inner nasal	–5.8 (–15.1, 3.5)	0.220	–24.4 (–43.1, –5.7)	0.011	–6.1 (–14.9, 2.6)	0.169	–0.7 (–8.9, 7.6)	0.874	7.4 (–4.8, 19.6)	0.236	–4.9 (–15.4, 5.6)	0.356	–5.1 (–13.0, 2.8)	0.205	0.5 (–11.3, 12.3)	0.930
3	CT at outer nasal	–3.5 (–11.7, 4.7)	0.405	–25.8 (–42.3, –9.4)	0.002	–4.5 (–12.3, 3.3)	0.257	0.7 (–6.6, 8.0)	0.845	4.0 (–6.8, 14.9)	0.465	–4.4 (–13.3, 4.5)	0.336	–4.4 (–11.5, 2.6)	0.220	0.0 (–10.4, 10.4)	0.996
4	CT at inner superior	–5.5 (–14.2, 3.3)	0.219	–20.9 (–39.3, –2.4)	0.027	–5.3 (–13.6, 3.0)	0.209	0.6 (–7.3, 8.5)	0.877	4.5 (–6.9, 15.9)	0.439	–5.9 (–16.1, 4.4)	0.260	–6.2 (–13.7, 1.3)	0.106	1.5 (–10.7, 13.7)	0.811
5	CT at outer superior	–6.4 (–14.4, 1.5)	0.111	–20.2 (–37.5, –2.8)	0.023	–6.1 (–13.7, 1.6)	0.120	–0.1 (–7.5, 7.4)	0.987	2.7 (–7.9, 13.4)	0.611	–4.8 (–14.3, 4.8)	0.327	–5.1 (–12.2, 2.0)	0.156	2.2 (–9.4, 13.9)	0.707
6	CT at inner temporal	–7.7 (–16.2, 0.9)	0.079	–16.4 (–34.8, 1.9)	0.079	–7.2 (–15.3, 1.0)	0.087	–1.1 (–9.0, 6.8)	0.780	6.7 (–4.8, 18.2)	0.253	–4.4 (–14.7, 5.9)	0.405	–3.0 (–10.5, 4.6)	0.440	1.3 (–10.1, 12.7)	0.818
7	CT at outer temporal	–7.2 (–14.7, 0.4)	0.062	–12.0 (–28.5, 4.5)	0.152	–6.7 (–13.9, 0.6)	0.071	–0.9 (–8.0, 6.2)	0.793	4.2 (–6.0, 14.4)	0.418	–4.6 (–13.8, 4.6)	0.328	–0.9 (–7.6, 5.8)	0.793	2.5 (–7.7, 12.8)	0.628
8	CT at inner inferior	–7.1 (–16.2, 2.1)	0.128	–18.7 (–38.6, 1.3)	0.066	–7.2 (–15.8, 1.5)	0.105	–0.8 (–9.2, 7.5)	0.844	7.9 (–4.3, 20.2)	0.205	–4.5 (–15.0, 6.0)	0.400	–2.5 (–10.4, 5.4)	0.539	2.5 (–9.1, 14.0)	0.674
9	CT at outer inferior	–7.8 (–16.7, 1.2)	0.090	–14.9 (–34.4, 4.6)	0.134	–8.0 (–16.5, 0.5)	0.066	–1.0 (–9.2, 7.1)	0.804	8.0 (–4.0, 20.1)	0.191	–3.8 (–13.8, 6.3)	0.462	–1.9 (–9.5, 5.8)	0.633	5.1 (–6.1, 16.2)	0.373
**Indians (238 eyes, 147 individuals)**																	
1	CT of SFCT (center)	31.7 (12.6, 50.9)	**0.001**	18.7 (1.1, 36.3)	0.038	19.8 (3.0, 36.6)	0.021	14.2 (–3.6, 32.0)	0.117	5.7 (–29.2, 40.5)	0.748	–6.0 (–27.4, 15.4)	0.581	–6.9 (–23.1, 9.4)	0.404	–0.0 (–18.2, 18.1)	0.996
2	CT at inner nasal	26.5 (10.7, 42.2)	**0.001**	25.7 (10.0, 41.4)	**0.002**	20.0 (6.7, 33.4)	**0.004**	12.0 (–4.0, 28.1)	0.139	–0.9 (–28.6, 26.8)	0.949	–9.5 (–28.2, 9.2)	0.315	–3.1 (–16.8, 10.6)	0.652	–7.8 (–23.4, 7.8)	0.324
3	CT at outer nasal	19.6 (5.4, 33.8)	0.007	22.1 (8.4, 35.8)	**0.002**	18.0 (5.9, 30.1)	**0.004**	9.4 (–4.6, 23.5)	0.188	–6.8 (–30.6, 16.9)	0.571	–10.1 (–26.1, 5.8)	0.212	–1.8 (–14.0, 10.4)	0.770	–2.7 (–15.8, 10.5)	0.689
4	CT at inner superior	21.3 (5.9, 36.7)	0.007	22.8 (5.5, 40.0)	0.01	12.6 (–0.5, 25.7)	0.058	12.2 (–2.7, 27.0)	0.107	7.8 (–19.2, 34.8)	0.570	–11.1 (–28.5, 6.3)	0.209	–3.8 (–17.0, 9.4)	0.572	–7.3 (–22.4, 7.8)	0.340
5	CT at outer superior	14.0 (–1.0, 29.0)	0.067	21.3 (3.6, 39.0)	0.018	8.6 (–4.6, 21.8)	0.2	9.4 (–4.2, 23.1)	0.174	5.0 (–21.5, 31.4)	0.711	–12.1 (–28.6, 4.3)	0.146	–2.4 (–15.0, 10.3)	0.711	–8.5 (–22.8, 5.8)	0.241
6	CT at inner temporal	25.3 (11.8, 38.8)	**< 0.001**	23.4 (7.3, 39.5)	**0.005**	17.2 (5.1, 29.3)	**0.006**	13.4 (–1.3, 28.2)	0.074	11.9 (–11.7, 35.6)	0.320	–7.3 (–24.3, 9.7)	0.398	–3.3 (–15.4, 8.8)	0.591	–6.3 (–19.8, 7.2)	0.355
7	CT at outer temporal	19.5 (6.9, 32.1)	**0.003**	22.1 (5.9, 38.3)	0.008	14.9 (3.2, 26.6)	0.013	13.0 (–0.4, 26.4)	0.057	12.5 (–8.4, 33.5)	0.239	–8.1 (–23.6, 7.4)	0.305	–1.0 (–12.5, 10.5)	0.863	–5.0 (–17.3, 7.2)	0.418
8	CT at inner inferior	23.7 (7.7, 39.7)	**0.004**	24.4 (8.9, 39.9)	**0.002**	19.3 (6.2, 32.5)	**0.004**	14.1 (–2.3, 30.6)	0.092	8.6 (–19.1, 36.4)	0.539	–7.5 (–27.1, 12.1)	0.449	0.5 (–13.2, 14.2)	0.944	–6.1 (–22.1, 10.0)	0.454
9	CT at outer inferior	21.5 (5.8, 37.2)	0.008	26.2 (10.9, 41.6)	**0.001**	21.8 (8.4, 35.1)	**0.002**	13.8 (–2.6, 30.1)	0.099	5.2 (–22.3, 32.8)	0.707	–9.3 (–28.9, 10.3)	0.352	6.2 (–8.0, 20.3)	0.391	–0.1 (–17.0, 16.9)	0.995

We conducted generalized estimating equation model regressing subfields of CT against genotypes of each genetic variant, adjusted on age, gender, axial length and refractive error. Both eye data were included clustering by individual. Beta coefficient is interpreted as the increase in mean CT per risk allele increasing. CT at mean nasal, mean of inner and outer nasal; CT at mean Superior, mean of inner and outer superior; CT

at mean temporal, mean of inner and outer temporal; CT at mean inferior, mean of inner and outer inferior. For each subfield of choroidal thickness, significant association p values were highlighted in bold based on the Bonferroni correction p value of 0.05/8=6.25E-3.

Supplementary Table 4. Association of AMD and CSC risk allele doses with subfoveal choroidal thickness (SFCT) in the three ethnic cohorts further adjusted on the time of OCT acquisition.

**SNPs**	**Effect allele/other allele**	**All cohorts (n = 1045/1788 eyes)**		**Chinese (n = 550/941 eyes)**		**Indian (n = 147/238 eyes)**		**Malay (n = 348/609 eyes)**	
		**β (95% CI)[a]**	**p value[b]**	**β (95% CI)[a]**	**p value[b]**	**β (95% CI)[a]**	**p value[b]**	**β (95% CI)[a]**	**p value[b]**
CFH									
rs800292	G/A	–4.8 (–12.5 to 3.0)	0.227	–6.6 (–18.1 to 4.9)	0.261	31.9 (13.0 to 50.9)	0.001**	–13.0 (–23.3 to –2.7)	0.014*
rs1061170	C/T	–4.2 (–15.4 to 7.1)	0.467	–23.2 (–49.7 to 3.3)	0.086	18.4 (1.2 to 35.6)	0.036*	–20.2 (–36.9 to –3.6)	0.017*
rs1329428	C/T	–5.1 (–12.5 to 2.3)	0.177	–6.8 (–17.7 to 4.0)	0.218	19.8 (3.4 to 36.3)	0.018*	–13.1 (–24.0 to –2.3)	0.017*
rs61818925	G/T	–2.1 (–9.1 to 4.9)	0.560	–1.7 (–11.8 to 8.4)	0.741	14.1 (–3.2 to 31.4)	0.109	–10.2 (–21.2 to 0.9)	0.072
VIPR2									
rs3793217	G/A	3.0 (–9.3 to 15.2)	0.637	11.1 (–4.9 to 27.0)	0.174	5.7 (–28.7 to 40.0)	0.746	–18.3 (–35.0 to –1.6)	0.032*
rs7782658	A/G	–5.7 (–15.0 to 3.6)	0.228	–7.4 (–20.7 to 5.9)	0.276	–5.9 (–26.8 to 15.0)	0.582	–5.9 (–19.6 to 7.7)	0.394
ARMS2									
rs10490924	T/G	–4.2 (–11.2 to 2.8)	0.240	–6.5 (–16.3 to 3.3)	0.193	–6.9 (–22.8 to 9.0)	0.396	0.8 (–9.9 to 11.5)	0.883
CETP									
rs3764261	A/C	6.9 (–2.3 to 16.1)	0.143	2.5 (–12.0 to 17.0)	0.736	–0.8 (–18.1 to 16.6)	0.932	7.4 (–8.0 to 22.\7)	0.346

[a]Beta coefficient is the increase in mean SFCT (μm) for each effect allele increase and is derived from generalized estimating equation model of SFCT against genotypes, adjusted for age, gender, axial length, refractive error, and time of OCT acquisition (AM or PM), with clustering by individual. OCT acquisition took place between 8.00 AM and 11.59 AM for the AM group and between 12.00 PM and 5.00 PM for the PM group.

[b]A Bonferroni corrected p value of 0.05/8=6.25E-3 was used as a cutoff for significance. Significant p values reaching this threshold were annotated with two asterisks. P values between 0.05 and 6.25E-3 were deemed marginally significant with a single asterisk symbol.

Supplementary Table 5. Pairwise interaction analysis for risk allele associations with SFCT between Chinese, Indian, and Malay cohorts.

	Indian / Chinese		Indian / Malay		Malay / Chinese		
**Risk alleles**	**β (95% CI)[a]**	**p**	**β (95% CI)[a]**	**p**	**β (95% CI)[a]**	**p**	**Overall interaction p**
CFH							
rs800292	37.8 (16.1 to 59.6)	0.001	44.3 (23.3 to 65.4)	<0.001	–6.5 (–22.2 to 9.1)	0.414	<0.001
rs1061170	42.3 (10.6 to 74.1)	0.009	39.0 (14.1 to 63.8)	0.002	3.4 (–28.4 to 35.2)	0.835	0.003
rs1329428	29.0 (9.2 to 48.8)	0.004	34.0 (14.4 to 53.6)	0.001	–5.0 (–20.5 to 10.5)	0.524	0.002
rs10922109	28.9 (9.1 to 48.7)	0.004	34.0 (14.4 to 53.6)	0.001	–5.1 (–20.5 to 10.4)	0.522	0.002
rs61818925	13.8 (–6.3 to 33.9)	0.178	21.8 (1.2 to 42.5)	0.038	–8.0 (–23.0 to 6.9)	0.292	0.113
VIPR2							
rs3793217	–4.5 (–42.6 to 33.5)	0.815	27.1 (–12.0 to 66.2)	0.174	–31.7 (–55.6 to –7.7)	0.010	0.031
rs7782658	2.8 (–22.4 to 28.0)	0.826	1.3 (–24.3 to 26.9)	0.921	1.5 (–17.9 to 21.0)	0.878	0.973
ARMS2							
rs10490924	–0.7 (–19.2 to 17.8)	0.939	–7.7 (–26.8 to 11.3)	0.425	7.0 (–7.9 to 21.9)	0.355	0.590
CETP							
rs3764261	0.3 (–22.4 to 23.0)	0.982	–4.4 (–28.0 to 19.2)	0.717	4.6 (–17.0 to 26.2)	0.675	0.902
rs2303790			Only 1 individual (Malay) with risk allele				

[a]Beta coefficient is the interaction coefficient between allele frequency and ethnicity i.e., the difference in the effect of each SNP on CT between pairs of ethnic groups
